# Associations between total, free and bioavailable 25-hydroxyvitamin D forms with adiponectin and irisin in maternal-neonatal pairs at birth from Greece

**DOI:** 10.3389/fendo.2024.1397869

**Published:** 2024-07-05

**Authors:** Tarek Ziad Arabi, Hana M. A. Fakhoury, Hani Tamim, Rene F. Chun, Martin Hewison, Fatme AlAnouti, Stefan Pilz, Cedric Annweiler, Georgios Tzimagiorgis, Costas Haitoglou, Spyridon N. Karras

**Affiliations:** ^1^ Department of Biochemistry and Molecular Medicine, College of Medicine, Alfaisal University, Riyadh, Saudi Arabia; ^2^ Department of Epidemiology and Biostatistics, College of Medicine, Alfaisal University, Riyadh, Saudi Arabia; ^3^ Department of Orthopaedic Surgery, David Geffen School of Medicine at University of California, Los Angeles (UCLA), Los Angeles, CA, United States; ^4^ Institute of Metabolism and Systems Research, University of Birmingham, Birmingham, United Kingdom; ^5^ Department of Public Health and Nutrition, College of Natural and Health Sciences, Zayed University, Abu Dhabi, United Arab Emirates; ^6^ ASPIRE Precision Medicine Research Institute Abu Dhabi, Abu Dhabi, United Arab Emirates; ^7^ Division of Endocrinology and Diabetology, Department of Internal Medicine, Medical University of Graz, Graz, Austria; ^8^ Department of Geriatric Medicine and Memory Clinic, Research Center on Autonomy and Longevity, Angers University Hospital, Angers, France; ^9^ Laboratory of Biological Chemistry, Medical School, Aristotle University, Thessaloniki, Greece

**Keywords:** vitamin D, adiponectin, irisin, neonatal, maternal, free vitamin D, bioavailable vitamin D2

## Abstract

**Background:**

Apart from the well-established skeletal effects, **v**itamin D has been explored as a secretagogue influencing various adipokines, including adiponectin and irisin. Recent evidence suggests that specific forms of 25-Hydroxyvitamin D (25(OHD), such as free and bioavailable 25(OH)D, may provide more accurate measurements of vitamin D status. The relationship between vitamin D status and serum irisin and adiponectin concentrations remains largely unexplored, particularly during pregnancy.

**Methods:**

We analyzed data from 67 healthy maternal-neonatal pairs from Northern Greece at birth. Biochemical and hormonal tests were conducted on each maternal-neonatal pair. The vitamin D forms were estimated using validated mathematical models. Subsequently, regression analyses were conducted to determine the association between the vitamin D forms and adipokine levels.

**Results:**

Bioavailable maternal 25(OH)D was inversely associated with neonatal irisin concentrations [β=-73.46 (-140.573 to -6.341), p=0.034]. No other associations were observed between maternal vitamin D status and neonatal adipokine concentrations.

**Conclusion:**

In conclusion, maternal bioavailable vitamin D concentrations are inversely associated with neonatal serum irisin concentrations, warranting further studies to evaluate the underlying mechanisms for this finding.

## 1 Introduction

Adipokines, hormones produced by adipocytes, exert diverse effects throughout the human body (1). Among these, adiponectin plays a pivotal role in promoting fatty acid oxidation, blunting gluconeogenesis, and mediating various anti-inflammatory effects (2). Adiponectin levels are influenced by gender and BMI, typically ranging between 2–20µg/ml (3). Numerous studies have underscored the significance of adiponectin levels in influencing neonatal outcomes. Yeung et al. reported a significant association between lower quartiles of neonatal adiponectin levels and an increased likelihood of preterm birth (4). Conversely, investigations of the association between maternal adiponectin concentrations and neonatal outcomes have yielded limited results (5, 6).

Irisin, a myokine and adipokine, is responsible for mediating several exercise-related metabolic changes and, potentially, exhibiting antineoplastic effects (7–12). Circulating irisin levels in humans are highly variable (0.01–2000 ng/ml) and influenced by various factors, including exercise, obesity, diet, diseases, and exposure to certain medications (13). Similar to the case of adiponectin, reduced cord blood concentrations of irisin have been associated with lower birthweight, but this correlation is not observed with maternal irisin levels (14). Additionally, diminished plasma irisin is independently linked with increased endothelial microparticles and endothelial progenitor cells, both of which are early markers of endothelial dysfunction, in preterm-born children (15). Conversely, elevated neonatal irisin levels have been linked to an increased risk of fetal macrosomia in a Chinese cohort (16).

It has been hypothesized that vitamin D may exert metabolic effects mediated through its effects on adipokines, including adiponectin (17). In a meta-analysis, Nikooyeh et al. concluded that vitamin D supplementation increases adiponectin concentrations in diabetic patients, hinting at its role as an adiponectin secretagogue (17). Similarly, vitamin D supplementation has been shown to increase irisin concentrations (18, 19), supporting the notion that vitamin D may have a broad effect in the regulation of adipokine secretion.

While total 25-hydroxyvitamin D [25(OH)D] is the most commonly used clinical measure of vitamin D status, debates still persist whether more accurate and clinically impactful measures are available (20). Existing in three forms in the body (21)—free, albumin-bound, and vitamin D binding protein-bound (VDBP)—25(OH)D’s biological activity is hypothesized to be primarily attributed to the free form (22). Hence, the free form could be a more accurate marker of vitamin D status in specific conditions, such as pregnancy (20). Our previous research has revealed a strong positive correlation of maternal VDBP with maternal adiponectin and irisin, while VDBP is associated with adiponectin but not irisin in neonates (23). Furthermore, we have demonstrated a maternal-neonatal relationship with free 25(OH)D levels, although no link to neonatal birth weight or anthropometry was identified (24).

To our knowledge, the associations between free and bioavailable (free with albumin-bound) forms of 25(OH)D with adiponectin and irisin levels have not been investigated. Unraveling these relationships holds the potential to shed light on the nature of vitamin D as a secretagogue of adipokines. Thus, our study aimed to analyze the associations between various forms of vitamin D and adipokine levels in maternal-neonatal pairs from the Mediterranean region.

## 2 Methods

### 2.1 Participants

Our study is a prospective study with a cross-sectional design consisting of 67 pairs of mothers and their newborns, as detailed previously (25). The study recruited fair-skinned women who delivered at term (between 37 and 42 weeks). Exclusion criteria encompassed conditions such as primary hyperparathyroidism, secondary osteoporosis, alcohol addiction, hyperthyroidism, nephritic syndrome, inflammatory bowel disease, rheumatoid arthritis, osteomalacia, obesity, gestational diabetes, or medication influencing calcium or vitamin D levels. Newborns classified as small for their gestational age or with severe congenital anomalies were also excluded. Ethical approval was obtained from the Bioethics Committee of Aristotle University in Thessaloniki, Greece (approval number 1/19–12-2011).

All participating mothers provided written consent prior to the study.

### 2.2 Demographic data

Upon registration, demographic and social attributes of mothers were recorded, including age, pre-pregnancy weight, gestational weeks, and number of prior live births. Maternal alcohol use during pregnancy was treated as a dichotomous variable, defined either as none (subdivided in never drinking alcohol or drinking alcohol but not during pregnancy) or light (1–2 units per week before pregnancy), moderate (3–6 units per week), or heavy (≥7 units per week or at any time during pregnancy).Tobacco consumption was recorded according by multiplying the number of packs of cigarettes smoked per day by the number of years the person has smoked.

### 2.3 Biochemical and hormonal tests

Blood samples from mothers were collected 30–60 minutes before delivery, and umbilical cord blood was collected post-clamping. Biochemical analyses included substances such as albumin, conducted as previously described (25, 26). The concentrations of 1,25(OH)2D, 25(OH)D2, and 25(OH)D3 were determined using liquid chromatography–tandem mass spectrometry, with a lower limit of quantification set at 0.5 ng/mL for each form. The total 25(OH)D was calculated by adding the concentrations of both forms, i.e. 25(OH)D2 plus 25(OH)D3. VDBP, irisin, and adiponectin were measured with enzyme-linked immunosorbent assay (ELISA) on a Synergy H1 Hybrid reader and Gen5 software (BioTek, Winooski, VT, USA). Detection limits for assays were 0.098 μg/ml for VDBP, 3.12 ng/ml for irisin, 0.039 μg/ml for adiponectin, and 5–100 ng/ml for 25(OH)D. Intra-assay and inter-assay variance were <8% and <10% for adiponectin and irisin, respectively.

### 2.4 Calculation of free and bioavailable vitamin D

Free and bioavailable 25(OH)D levels in both mothers and newborns were estimated using a mathematical model based on prior research by Chun et al. (27). Observed values of VDBP (μM), albumin (μM), 25(OH)D (nM), and 1,25(OH)2D (nM) from the samples were fed into a MATLAB script, which produced estimates of free and bioavailable 25(OH)D. Serum free 25(OH)D levels were converted from nM to pg/ml by multiplying 0.4166 × 10^3 (20).

### 2.5 Statistical analysis

Data was analyzed using the Statistical Package for Social Sciences (SPSS, version 28, IBM Corp., Armonk, NY). Descriptive statistics included numbers, percentages, means ± standard deviations for categorical and continuous variables, respectively. Associations between measures of vitamin D status and adipokines were calculated by univariate and multivariate linear regression analyses considering potential confounding factors such as maternal height, pre-pregnancy BMI, at-term BMI, and weeks of gestation (28). Results were expressed as beta coefficients (β) and 95% confidence intervals (CI). A p-value < 0.05 was considered statistically significant.

## 3 Results

### 3.1 Patient characteristics

A total of 67 pairs of mothers and their newborns are included in the current analysis. The average maternal age in our study cohort was 31.75 ± 6.11 years, and the gestational age was 38.83 ± 1.59 weeks. Of the participating mothers, 43 (69.4%) were nulliparous prior to the study, 14 (22.2%) had attained advanced education, 7 (11.1%) reported alcohol consumption during pregnancy, and 10 (15.9%) acknowledged smoking during pregnancy. A comprehensive overview of the study population is presented in **Table 1**.

**Table 1:** Sociodemographic and characteristics of the study population.

n= 67	Mean ± SD/N (%)
**Age (y)**	31.75 ± 6.11
**Weight pre-pregnancy (kg)**	67.01 ± 14.36
**Weight term (kg)**	80.74 ± 13.82
**Weeks of gestation**	38.83 ± 1.59
Previous live births	
0	43 (69.4%)
1	9 (14.5%)
2	8 (12.9%)
3	1 (1.6%)
4	1 (1.6%)
Missing	5
Educational level	
Standard	49 (77.8%)
Advanced	14 (22.2%)
Missing	4
Alcohol	
Consumption	56 (88.9%)
No consumption	7 (11.1%)
Missing	4
Smoking	
None	53 (84.1%)
Smoked	10 (15.9%)
Missing	4

### 3.2 Association between maternal vitamin D status and neonatal adipokine levels

**Figure 1:**
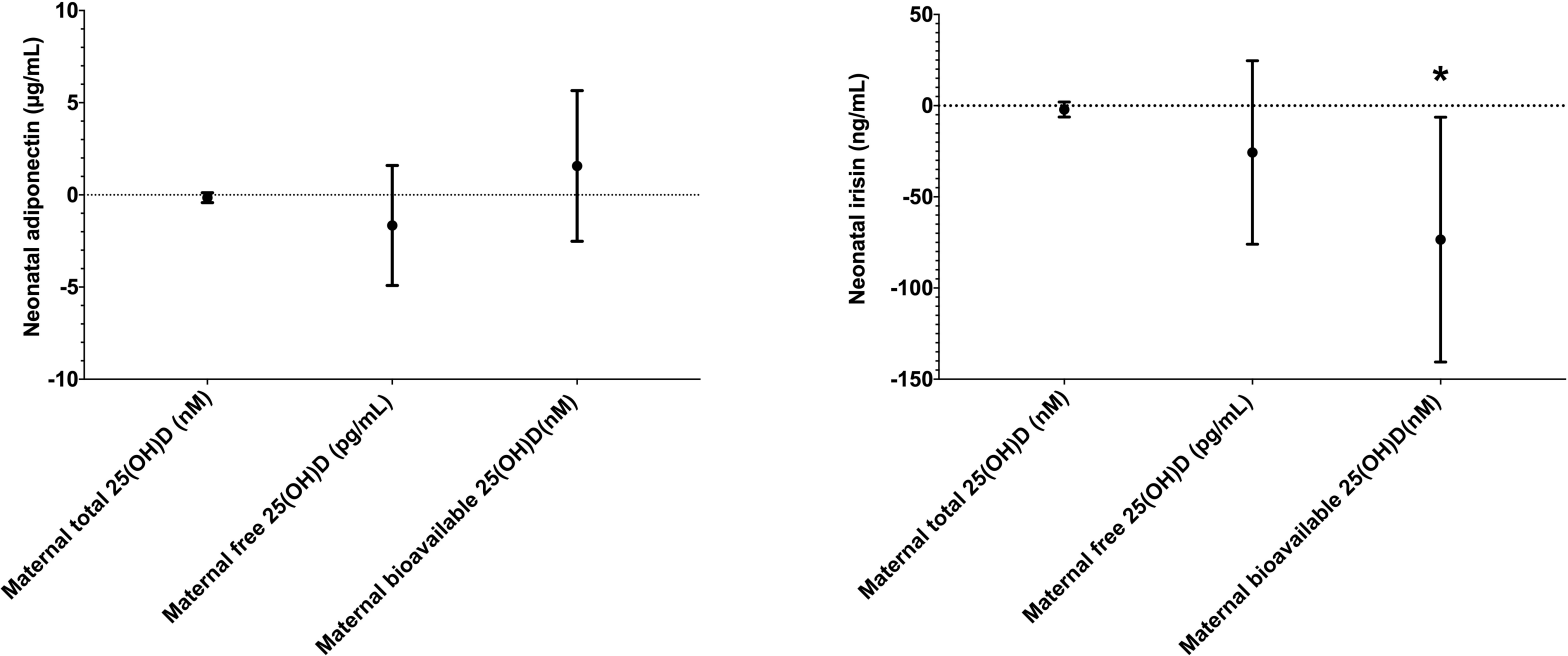
The association between maternal 25(OH)D forms and neonatal adiponectin (left) and irisin (right) in multivariate analysis. * indicates statistical significance.

Initially, we investigated the relationship between various maternal 25(OH)D forms and neonatal adipokine levels. Univariate and multivariate analyses revealed no associations between any 25(OH)D forms and neonatal adiponectin concentrations (**Table 2**). Similarly, maternal total and free 25(OH)D levels were not associated with neonatal irisin levels. However, bioavailable 25(OH)D, showed an inverse association with neonatal irisin in univariate analysis [β = -56.257 (-106.227 to -6.288), p = 0.028]. This relationship became more pronounced in multivariate analysis [β = -73.457 (-140.573 to -6.341), p = 0.034] (**Figure 1**).

**Table 2:** Relationship between maternal 25(OH)D forms and neonatal adipokine concentrations.

	Maternal total 25(OH)D (nM)		Maternal free 25(OH)D (pg/mL)		Maternal bioavailable 25 (OH)D(nM)	
	Univariate β (95% CI)	Multivariate β (95% CI)	Univariate β (95% CI)	Multivariate β (95% CI)	Univariate β (95% CI)	Multivariate β (95% CI)
Neonatal adiponectin (µg/mL)	0.037 (-0.188, 0.261)	-0.153 (-0.420, 0.114)	0.116 (-2.510, 2.743)	-1.660 (-4.913, 1.594)	1.279 (-2.428, 4.985)	1.568 (-2.518, 5.654)
	0.745	0.251	0.930	0.307	0.493	0.440
Neonatal irisin (ng/mL)	0.238 (-2.794, 3.269)	-2.128 (-6.258, 2.001)	6.030 (-28.626, 40.686)	-25.671 (-75.977, 24.635)	-56.257 (-106.227, -6.288)	-73.457 (-140.573, -6.341)
	0.875	0.295	0.726	0.299	**0.028**	**0.034**

Bolded values are statistically significant.

### 3.3 Exploration of intrarelationships between 25(OH)D forms and adipokines

Subsequently, we analyzed the intrarelationships between 25(OH)D forms and adipokines in the mothers and neonates. We found no association between the levels of maternal 25(OH)D forms with maternal adipokines. Similar results were also seen between neonatal 25(OH)D and adipokine concentrations **Table 3**.

**Table 3:** Intrarelationships between vitamin D status and adipokine levels in mothers and neonates.

	Maternal			Neonatal			
	Total 25(OH)D (nM)	Free 25(OH)D (pg/mL)	Bioavailable 25(OH)D(nM)		Total 25(OH)D (nM) baby	Free 25(OH)D (pg/mL)	Bioavailable 25(OH)D (nM)
**Maternal adiponectin (µg/mL)**	0.021 (-0.021, 0.064)	0.019 (-0.449, 0.487)	-0.036 (-0.794, 0.721)	**Neonatal adiponectin (µg/mL)**	0.115 (-0.060, 0.289)	-0.473 (-1.865, 0.918)	-0.598 (-2.193, 0.998)
	0.318	0.936	0.924		0.193	0.499	0.456
**Maternal irisin (ng/mL)**	-0.631 (-6.451, 5.189)	-30.952 (-94.603, 32.699)	-77.373 (-171.302, 16.556)	**Neonatal irisin (ng/mL)**	0.409 (-1.783, 2.602)	-5.402 (-21.928, 11.124)	-2.580 (-22.950, 17.791)
	0.829	0.334	0.105		0.708	0.512	0.799

## 4 Discussion

In this study, we investigated the associations between various forms of 25(OH)D and adipokine levels in maternal-neonatal pairs. Interestingly, we observed a significant inverse association between maternal bioavailable 25(OH)D and neonatal irisin levels, highlighting a potential role of this specific form of vitamin D in regulating neonatal adipokines. We observed no intrarelationships between maternal and neonatal 25(OH)D forms and adipokine levels.

The interplay between vitamin D and adipokines remains a topic of considerable debate. In the general population, vitamin D supplementation generally does not lead to increased adiponectin levels (17). However, subgroup analyses have suggested a positive effect among diabetic individuals and those undergoing daily supplementation (17). To our knowledge, the influence of maternal vitamin D on neonatal adiponectin has not been previously studied, and our findings revealed no association between maternal vitamin D forms and neonatal adiponectin.

Similarly, the connection between vitamin D and irisin is not well-established, with conflicting findings in various studies. For example, Prader–Willi syndrome patients who do not receive vitamin D supplementations have significantly lower irisin levels than those who do (29). In a double-blind, randomized clinical trial, vitamin D supplementation significantly increased irisin serum levels in overweight and obese type 2 diabetics (30). However, serum irisin levels are negatively correlated with 25(OH)D in Charcot-Marie-Tooth patients (31). Similarly, type 1 diabetes mellitus patients demonstrate a similar negative association (32). Other studies have also revealed no association between the two parameters (33). Our study found no association of total and free 25(OH)D with neonatal irisin levels. However, maternal bioavailable 25(OH)D is independently and inversely associated with neonatal irisin concentrations. This observation warrants further investigation, as this study is the first to explore the relationship between maternal vitamin D status and neonatal irisin. The reason why bioavailable 25(OH)D is negatively associated with neonatal irisin concentrations while the free form is not, remains unclear. However, this finding is in accordance with our previous results, where maternal VDBP manifested significant associations with maternal adiponectin and irisin, whereas neonatal VDBP was associated with neonatal adiponectin and irisin, although the later association was not significant. It becomes evident that non-bounds 25(OH)D forms, in this case maternal bioavailable forms, may exert reverse regulatory effects compared to bound forms and VBDP, implying a diverse metabolic role of free and bound vitamin D forms on adipokine concentrations. Further studies are needed to verify our findings and understand the relationship between bioavailable vitamin D forms and irisin.

The impact of irisin levels on neonatal outcomes presents divergent findings in the literature. In our study, we observed a correlation between maternal bioavailable 25(OH)D and reduced neonatal irisin. Some studies have reported that elevated irisin levels are linked to endothelial dysfunction in pre-term children (15). It could be hypothesized that maternal bioavailable 25(OH)D may provide a protective effect against endothelial dysfunction. However, contrasting results have been reported, with other studies indicating an inverse association between irisin levels and the risk of fetal macrosomia (16). The conflicting evidence in existing literature highlights the imperative need for future studies to untangle the intricate role of adipokines, particularly irisin, in neonatal outcomes.

This study has some limitations. A significant constraint is its single-center nature, which potentially limits the generalizability of our findings, i.e. a limited external validity. Variations in 25(OH)D levels due to differences in geographic locations and seasonal changes are well-documented (20). Due to its homogenous population and single location, these factors were not taken into consideration. Furthermore, there are several factors that can influence adipokine levels, such as exercise levels (34), that were not examined in our study. Our observational design also precludes inferring causality from the data. Additionally, the study might be susceptible to statistical type I errors (false positive findings) due to multiple testing. As our statistical analysis plan was however based on hypotheses derived from previous investigations by others, we refrained from adjusting for multiple testing.

In conclusion, our findings indicate that maternal bioavailable 25(OH)D is associated with reduced neonatal irisin concentrations, while total and free maternal 25(OH)D have no impact on neonatal adipokines. Intrarelationships between vitamin D forms and adipokine levels were not observed. The potential protective effects of bioavailable 25(OH)D against harmful irisin effects warrant further investigations. Future studies should explore the underlying mechanisms by which maternal bioavailable 25(OH)D regulates neonatal irisin levels. Additionally, investigating this interplay in diverse populations or at-risk groups like mothers with vitamin D deficiency could provide valuable insights.

## Data availability statement

The raw data supporting the conclusions of this article will be made available by the authors, without undue reservation.

## Ethics statement

The studies involving humans were approved by Bioethics Committee of Aristotle University in Thessaloniki. The studies were conducted in accordance with the local legislation and institutional requirements. Written informed consent for participation in this study was provided by the participants’ legal guardians/next of kin.

## Author contributions

TA: Methodology, Validation, Writing – original draft, Writing – review & editing. HF: Data curation, Visualization, Writing – original draft, Writing – review & editing. HT: Formal analysis, Visualization, Writing – original draft, Writing – review & editing. RC: Data curation, Validation, Writing – original draft, Writing – review & editing. MH: Investigation, Validation, Writing – review & editing. FA: Resources, Validation, Writing – review & editing. SP: Resources, Validation, Writing – review & editing. CA: Resources, Validation, Writing – review & editing. GT: Resources, Validation, Writing – review & editing. CH: Resources, Validation, Writing – review & editing. SK: Funding acquisition, Investigation, Methodology, Project administration, Resources, Supervision, Validation, Writing – original draft, Writing – review & editing.
